# Best Vitelliform Macular Dystrophy Natural History Study Report 1: Clinical Features and Genetic Findings

**DOI:** 10.1016/j.ophtha.2024.01.027

**Published:** 2024-07

**Authors:** Yannik Laich, Michalis Georgiou, Kaoru Fujinami, Malena Daich Varela, Yu Fujinami-Yokokawa, Shaima Awadh Hashem, Thales Antonio Cabral de Guimaraes, Omar A. Mahroo, Andrew R. Webster, Michel Michaelides

**Affiliations:** 1 Moorfields Eye Hospital, London, United Kingdom; 2 UCL Institute of Ophthalmology, University College London, London, United Kingdom; 3 Eye Center, Faculty of Medicine, University of Freiburg, Freiburg, Germany; 4 Jones Eye Institute, University of Arkansas for Medical Sciences, Little Rock, Arkansas; 5 Laboratory of Visual Physiology, Division of Vision Research, National Institute of Sensory Organs, NHO Tokyo Medical Center, Tokyo, Japan; 6 Department of Health Policy and Management, Keio University School of Medicine, Tokyo, Japan

**Keywords:** Best vitelliform macular dystrophy, Genetics, Natural history, Retinal dystrophy

## Abstract

**Purpose:**

To analyze the genetic findings, clinical spectrum, and natural history of Best vitelliform macular dystrophy (BVMD) in a cohort of 222 children and adults.

**Design:**

Single-center retrospective, consecutive, observational study.

**Participants:**

Patients with a clinical diagnosis of BVMD from pedigrees with a likely disease-causing monoallelic sequence variant in the *BEST1* gene.

**Methods:**

Data were extracted from electronic and physical case notes. Electrophysiologic assessment and molecular genetic testing were analyzed.

**Main Outcome Measures:**

Molecular genetic test findings and clinical findings including best-corrected visual acuity (BCVA), choroidal neovascularization (CNV) rates, and electrophysiologic parameters.

**Results:**

Two hundred twenty-two patients from 141 families were identified harboring 69 *BEST1* variants. Mean age at presentation was 26.8 years (range, 1.3–84.8 years) and most patients (61.5%) demonstrated deterioration of central vision. Major funduscopic findings included 128 eyes (30.6%) with yellow vitelliform lesions, 78 eyes (18.7%) with atrophic changes, 49 eyes (11.7%) with fibrotic changes, 48 eyes (11.5%) with mild pigmentary changes, and 43 eyes (10.3%) showing a vitelliruptive appearance. Mean BCVA was 0.37 logarithm of the minimum angle of resolution (logMAR; Snellen equivalent, 20/47) for the right eye and 0.33 logMAR (Snellen equivalent, 20/43) for the left eye at presentation, with a mean annual loss rate of 0.013 logMAR and 0.009 logMAR, respectively, over a mean follow-up of 9.7 years. Thirty-seven patients (17.3%) received a diagnosis of CNV over a mean follow-up of 8.0 years. Eyes with CNV that received treatment with an anti–vascular endothelial growth factor (VEGF) agent showed better mean BCVA compared with eyes that were not treated with an anti-VEGF agent (0.28 logMAR [Snellen equivalent, 20/38] vs. 0.62 logMAR [Snellen equivalent, 20/83]). Most eyes exhibited a hyperopic refractive error (78.7%), and 13 patients (6.1%) received a diagnosis of amblyopia. Among the 3 most common variants, p.(Ala243Val) was associated with a later age of onset, better age-adjusted BCVA, and less advanced Gass stages compared with p.(Arg218Cys) and p.(Arg218His).

**Conclusions:**

BVMD shows a wide spectrum of phenotypic variability. The disease is very slowly progressive, and the observed phenotype–genotype correlations allow for more accurate prognostication and counselling.

**Financial Disclosure(s):**

Proprietary or commercial disclosure may be found in the Footnotes and Disclosures at the end of this article.

Best vitelliform macular dystrophy (BVMD; Online Mendelian Inheritance in Man identifier, 153700) is an autosomal dominant inherited retinal disease. Although considered a rare genetic disorder, it is the second most common macular dystrophy and the most frequent autosomal dominant macular dystrophy.^1^

BVMD is caused by monoallelic variants in the *BEST1* gene, which is located on chromosome 11q12.3 and encodes the integral membrane protein bestrophin 1, a chloride channel primarily found on the basolateral plasma membrane of the retinal pigment epithelium.^2^, ^3^, ^4^ The retinal degeneration can assume different anatomic configurations over time, as described by Gass^5^ in a 5-stage classification system: stage 1, previtelliform; stage 2, vitelliform; stage 3, pseudohypopyon; stage 4, vitelliruptive; and stage 5, atrophy or fibrosis. Electrooculography plays an important role in the diagnosis of BVMD, with a reduced light peak resulting in a lowered Arden ratio.^6^ However, genetic testing is crucial to confirm the diagnosis by identifying a pathogenic monoallelic variant in *BEST1*.

Robust and detailed natural history data from a large cohort of genetically confirmed patients with BVMD are lacking. Such data will provide more accurate prognostic information with implications to improve patient counseling and clinical trial design. To address this gap, this study aimed to characterize the clinical phenotype, molecular genotype, and natural history of BVMD comprehensively using a large single-center cohort.

## Methods

This retrospective cohort study conformed to the tenets of the Declaration of Helsinki and was approved by the Moorfields Eye Hospital ethics committee. All patients included in this database had provided informed consent previously.

### Patient Identification

All patients with a monoallelic variant in *BEST1* and a clinical diagnosis of BVMD or a clinical diagnosis of BVMD with at least 1 family member showing positive genetic test results for *BEST1* in a tertiary referral center (Moorfields Eye Hospital, London, United Kingdom) were reviewed. The patients were identified using in-house databases (OpenEyes and MagicXPA 3.3; Moorfields Eye Hospital). Subsequently, information was extracted from electronic health care records and physical case notes. Patients with other concurrent ocular pathologic features were excluded.

### Clinical Data

Clinical data extracted included presenting symptoms, best-corrected visual acuity (BCVA), refraction, and slit-lamp biomicroscopy and funduscopy findings. BCVA data at the initial presentation and at the most recent follow-up (final) visit were analyzed. When necessary, Snellen and decimal acuity were converted into logarithm of the minimum angle of resolution (logMAR) values.^6^^,^^7^ The definition for reduced vision of worse than 0.2 logMAR (Snellen equivalent, 20/32) from the United Kingdom school screening was used.^8^ Mean annual progression rate for BCVA loss was calculated per eye by subtraction of BCVA at first visit from BCVA at the last visit divided by the specific follow-up period for every patient. Amblyopia was defined clinically according to the American Academy of Ophthalmology as a difference in BCVA of 2 lines or more (0.2 logMAR or more) between eyes.^9^ Myopic refractive errors were classified as follows: low myopia, –0.50 diopter (D) to –6.00 D; and high myopia, –6.00 D or less. Hyperopic errors were classified as follows: low, 0.25 D to 2.25 D; moderate, 2.25 D to 5.25 D; and high, 5.25 D or more.^10^^,^^11^ Color or pseudocolor fundus photographs were obtained with either the Optos ultra-widefield camera (Optos PLC) or the TRC-50LA retinal fundus camera (Topcon). Fundus appearance was graded in the previously described stages of BVMD: stage 1, previtelliform; stage 2, vitelliform; stage 3, pseudohypopyon; stage 4, vitelliruptive; and stage 5, atrophy or fibrosis. The presence of unifocal or multifocal fundus changes also was noted.

### Electrophysiologic Testing

Electrophysiologic testing included electrooculography performed according to the standards of the International Society for Clinical Electrophysiology of Vision.^12^ A light peak-to-dark trough ratio of 1.5 or less was considered suggestive of BVMD.^6^

### Genetic Testing and Analysis

As part of routine clinical diagnostics, a combination of targeted Sanger sequencing, next-generation sequencing, sequencing panels of retinal dystrophy genes, whole-exome sequencing, and whole-genome sequencing was used to identify variants in the *BEST1* gene. All recruited patients were reassessed for their detected variants as described in the Supplemental Methods (available at www.aaojournal.org).

### Genotype–Phenotype Correlation

Patients with the most prevalent variants (for which at least 8 patients’ data are available) were selected for genotype–phenotype correlation analysis and were compared for age at onset, age-adjusted BCVA, and distribution of Gass stages. Age adjustment of BCVA was required because of different age distributions between the groups and the correlation between BCVA and age described in this cohort. We calculated age-adjusted BCVA by adding or subtracting the mean annual progression rate multiplied by the age difference between the actual age when BCVA was measured and a standardized age of 40 years for every eye.

### Statistical Analysis

Statistical analysis was performed using Prism version 8.0.2 software (GraphPad Software). The threshold for significance for all statistical tests was set at a *P* value of less than 0.05.

## Results

### Patient Characteristics

Two hundred twenty-two patients (127 male patients [57.2%]) from 141 pedigrees met the genotype and phenotype inclusion criteria. One patient was excluded from analysis of clinical findings and imaging after having a central retinal artery occlusion consecutively in both eyes before the first visit. One eye was excluded from BCVA analysis after retinal detachment with macular involvement, and 1 eye was excluded from BCVA analysis while having a corneal ulcer. Thirteen eyes were excluded from BCVA analysis because of amblyopia, and for 3 patients, BCVA at baseline was decreased because of their young age (related to ability to comply with testing), and they exhibited improved BCVA of more than 0.2 logMAR at subsequent visits. We identified 374 patients from an electronic patient letter database with a presumed diagnosis of BVMD who were not included in this cohort because they did not meet the genetic inclusion criteria. A proportion of these patients presumably did not have BVMD resulting from *BEST1*, including those who in fact may have acquired disease or may have vitelliform maculopathy resulting from one of many other genes. Historical limitations have restricted the availability of genetic testing, as well as instances of loss to follow-up before genetic testing could be administered. Patients seen in nongenetic clinics may not have been offered or had access to genetic testing, and some patients or their families declined genetic testing. For patients who underwent testing, the failure to identify a sequence variant in *BEST1* also led to exclusion from this study. Overall, the cohort of 222 patients from pedigrees with a likely disease-causing sequence variant represents 37.2% of all identified patients with a presumed diagnosis of BVMD.

### Age at Presentation and Symptoms of Onset

**Figure 1:**
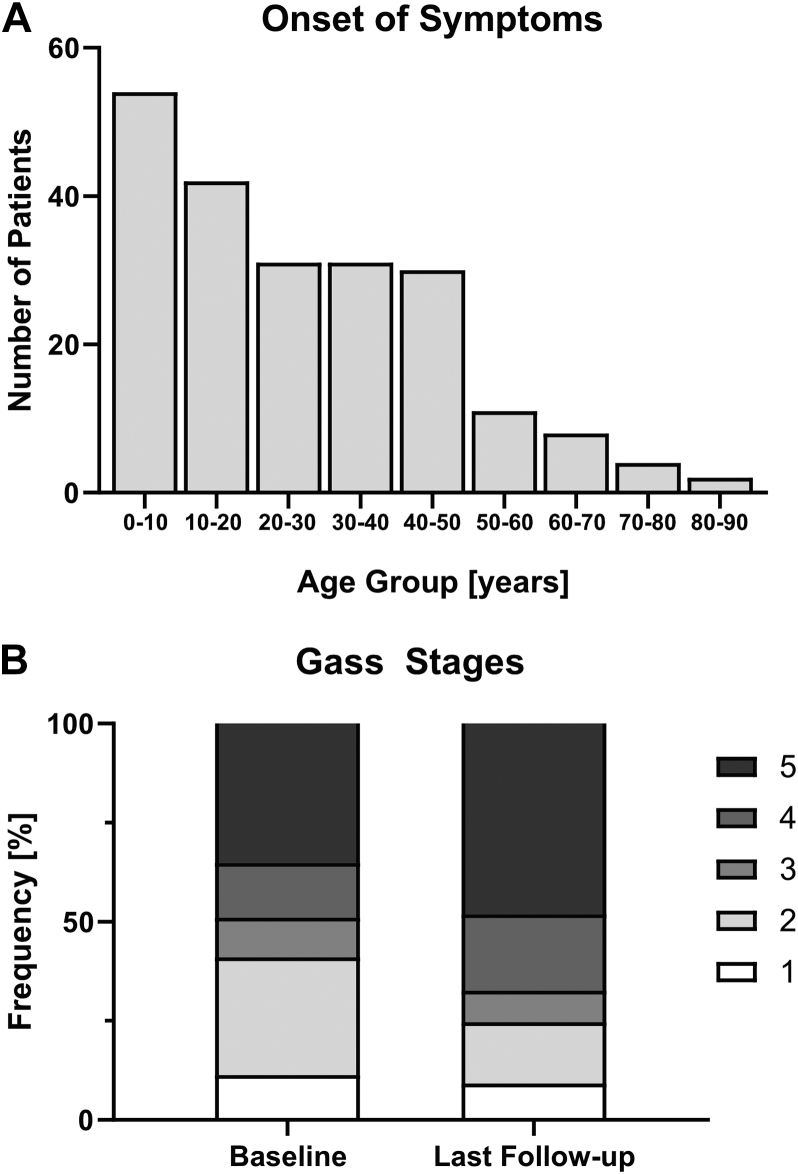
Bar graphs showing (**A**) number of patients by age group at the age of onset, with a peak having onset in childhood, and (**B**) distribution of Gass stages at baseline and last follow-up.

Age at presentation was documented for 213 patients (96.0%). Mean age ± standard deviation (SD) at presentation was 26.8 ± 19.1 years (range, 1.3–84.8 years), with most patients presenting in childhood or early adulthood (Fig 1A). At presentation, 131 patients (61.5%) demonstrated a deterioration of vision, 26 patients (12.2%) were asymptomatic and had been referred because of family history or incidental findings on annual examination, 10 patients (4.7%) reported distorted vision, and 4 patients (1.9%) reported the perception of a scotoma. For 33 patients (15.5%), no initial symptoms were documented. Table 1 summarizes a complete list of the presenting symptoms. No patient demonstrated acute angle-closure glaucoma at presentation. Six patients (2.8%) with chronic angle closure underwent prophylactic peripheral yttrium–aluminum–garnet laser iridotomy, 5 of them bilaterally, 1 of them unilaterally.

**Table 1:** Presenting Symptoms

Symptom	No. of Patients (%)
Deterioration of vision	131 (61.5)
Asymptomatic	26 (12.2)
Distorted vision	10 (4.7)
Perception of a scotoma	4 (1.9)
Photopsia	2 (0.9)
Fluctuation of vision	1 (0.5)
Clumsiness	1 (0.5)
Presbyopic symptoms	1 (0.5)
Symptoms of eye strain	1 (0.5)
Vitreous hemorrhage	1 (0.5)
Difference of vision when used monocularly	1 (0.5)
Issues with night vision	1 (0.5)
Not documented	33 (15.5)

### Visual Acuity and Refraction

**Figure 2:**
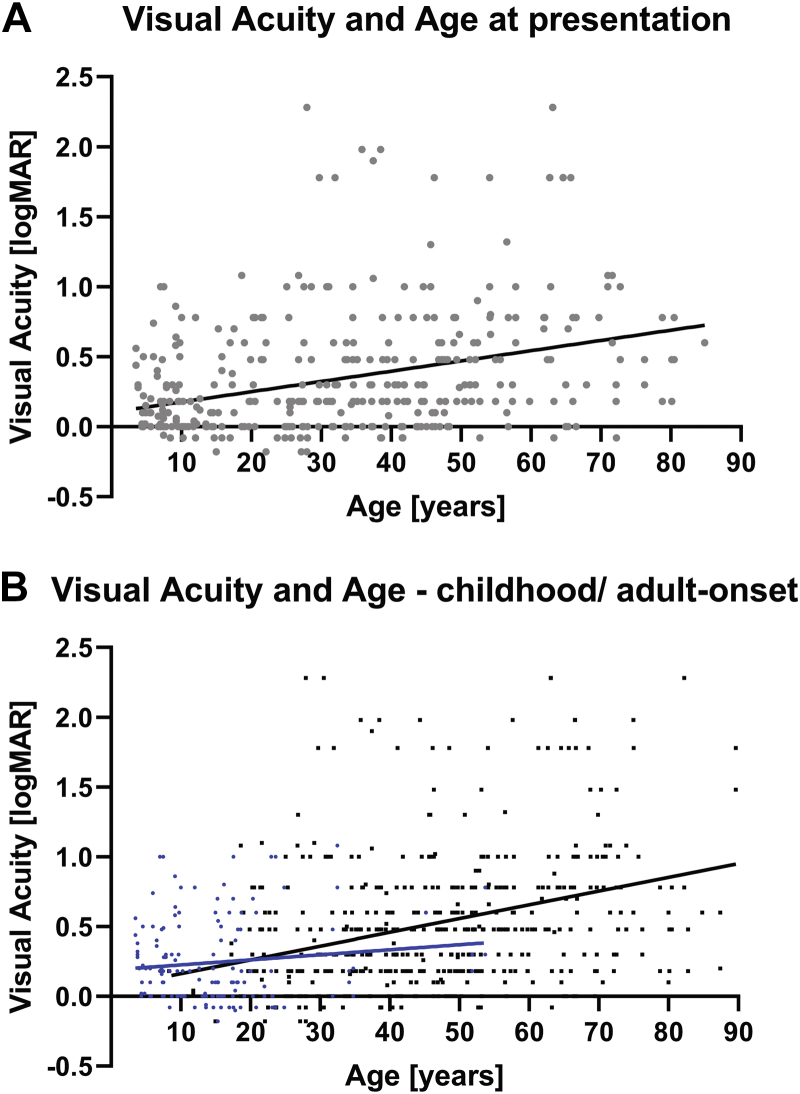
Scatterplots showing (**A**) best-corrected visual acuity at baseline against age with linear regression showing the line of best fit and (**B**) visual acuity at baseline and last follow-up for the childhood-onset (blue) and adult-onset (black) groups plotted against age with linear regression showing the lines of best fit. logMAR = logarithm of the minimum angle of resolution.

Two hundred twelve patients had a documented BCVA for at least 1 eye at presentation. Mean ± SD BCVA was 0.37 ± 0.47 logMAR (Snellen equivalent, mean, 20/47; range, –0.18 to 2.28 logMAR [Snellen equivalent, 20/13–20/3811]) for the right eye and 0.33 ± 0.42 logMAR (Snellen equivalent, mean, 20/43; range, –0.18 to 2.28 logMAR [Snellen equivalent, 20/13–20/3811]) for the left eye at presentation. Baseline BCVA was highly variable among patients, but no significant interocular difference was found (*t* = 0.72; *P* = 0.47, paired *t* test). Data from both eyes were pooled and plotted against age (Fig 2A). A statistically significant weak correlation was found between BCVA and age at presentation (*r* = 0.33; *P* < 0.0001, Pearson correlation coefficient).

Refraction data from 235 eyes of 119 patients were included in the analysis. The spherical equivalent was calculated, and refractive errors were classified. Thirty-four eyes (14.5%) were found to have high hyperopia, 67 eyes (28.5%) were found to have moderate hyperopia, and 84 eyes (35.7%) were found to have low hyperopia. Eleven eyes (4.7%) were emmetropic and 39 eyes exhibited low myopia (16.6%). No patient was found to have high myopia. Of the 213 patients with clinical information, amblyopia was diagnosed in 13 patients (6.1%), with 1 patient successfully treated with occlusion therapy. The cause for amblyopia was strabismus in 8 patients, refractive error in 3 patients, and not documented for 2 patients.

### Funduscopic Findings and Color Fundus Photography

Funduscopic description or color fundus photographs were available for 418 eyes from 209 patients. One hundred twenty-eight eyes (30.6%) exhibited yellow vitelliform lesions, followed by 78 eyes (18.7%) with atrophic changes and 49 eyes (11.7%) with fibrotic changes. Forty-eight eyes (11.5%) showed only mild pigmentary changes and 43 eyes (10.3%) were found to have a vitelliruptive appearance. Twenty-three eyes (5.5%) demonstrated subretinal fluid on funduscopy and 22 eyes (5.3%) showed a pseudohypopyon appearance. Twenty-one eyes (5.0%) were described without any pathologic changes and 6 eyes (1.4%) exhibited retinal hemorrhages. In 7 patients (3.3%), morphologic changes were limited to 1 eye, whereas the unaffected eye did not exhibit any changes. Of 397 eyes exhibiting changes, 339 eyes (85.4%) showed unifocal features, whereas in 58 eyes (14.6%), multifocal changes were observed. Of 31 patients with multifocal changes, 27 patients (87.1%) exhibited bilateral multifocal disease. Table S2 (available at www.aaojournal.org) presents a list of the peripheral retinal findings.

### Choroidal Neovascularization

Of the 213 patients with clinical information, a total of 37 patients (17.3%) had received a clinical diagnosis of choroidal neovascularization (CNV), of whom 28 patients (13.1%) had a unilateral occurrence and 9 patients (4.2%) were affected bilaterally over a mean course of follow-up of 8.0 years (range, 0–55 years). Mean ± SD BCVA at the last follow-up was 0.44 ± 0.42 logMAR (Snellen equivalent, mean, 20/55; range, 0.00–2.28 logMAR [Snellen equivalent, 20/20–20/3811]) for eyes with a diagnosis of CNV (mean age at last follow-up, 34.4 years) and 0.47 ± 0.52 logMAR (Snellen equivalent, mean, 20/59; range, –0.20 to 3.00 logMAR [Snellen equivalent, 20/13–20/20 000]) for eyes without a diagnosis of CNV (mean age at last follow-up, 42.8 years), with no significant difference between the groups (*t* = 0.37; *P* = 0.71, unpaired *t* test with Welch’s correction).

Of the 46 eyes with a diagnosis of CNV, 24 eyes (52.2%) were treated with at least 1 intravitreal injection of an anti–vascular endothelial growth factor (VEGF) agent. Mean ± SD BCVA at the last follow-up was 0.28 ± 0.25 logMAR (Snellen equivalent, mean, 20/38; range, 0.00–0.78 logMAR [Snellen equivalent, 20/20–20/121]) for eyes that were treated with an anti-VEGF agent and 0.62 ± 0.48 logMAR (Snellen equivalent, mean, 20/83; range, 0.00–2.28 logMAR [Snellen equivalent, 20/20–20/3811]) for eyes that were not treated with an anti-VEGF agent, with a significantly better mean BCVA in the group that received anti-VEGF therapy (*t* = 3.0; *P* = 0.005, unpaired *t* test with Welch’s correction). Mean ± SD BCVA at the time of diagnosis of CNV was available for 35 eyes and did not reveal a significant difference between groups: eyes treated with an anti-VEGF agent, 0.60 ± 0.27 logMAR (Snellen equivalent, mean, 20/80; range, 0.16 to 1.22 logMAR [Snellen equivalent, 20/29–20/332]) versus eyes not treated with an anti-VEGF agent, 0.79 ± 0.47 logMAR (Snellen equivalent, mean, 20/123; range, 0.10–1.68 logMAR [Snellen equivalent, 20/25–20/957]; *t* = 1.4; *P* = 0.18, unpaired *t* test with Welch’s correction). Mean age at CNV occurrence was similar in both groups: eyes treated with an anti-VEGF agent, 25.5 ± 16.6 years (range, 6.0–58.8 years) versus eyes not treated with an anti-VEGF agent, 27.5 ± 21.7 years (range, 4.3–73.1 years; *t* = 0.35; *P* = 0.73, unpaired *t* test with Welch’s correction). CNV occurrence since the diagnosis of BVMD also was similar in both groups: eyes treated with an anti-VEGF agent, 5.9 ± 10.0 years (range, 0–40.0 years) versus eyes not treated with an anti-VEGF agent, 8.9 ± 15.1 years (range, 0–63.1 years; *t* = 0.81; *P* = 0.42, unpaired *t* test with Welch’s correction). Eighteen eyes that did not receive an anti-VEGF agent had a documented reason why no anti-VEGF treatment was administered: 7 eyes (38.9%) had fibrotic changes or chronic edema, hence the treating clinician considered that there was no potential for improvement with therapy; 5 eyes (27.8%) received a diagnosis before anti-VEGF therapy was available; 4 eyes (22.2%) did not reveal significant CNV activity at the time of diagnosis, 1 eye (5.6%) had an extrafoveal location of the CNV, and in 1 eye (5.6%), the parents of the patient declined the treatment and opted for observation. The total number of injections was available for 20 eyes and ranged from 1 to 11 injections, with a mean ± SD of 2.95 ± 2.50 injections per eye. Two adverse events after injection were documented: a small vitreous hemorrhage that resolved spontaneously and transient vision loss with photopsia without any abnormality on ophthalmologic examination.

### Longitudinal Analysis of Visual Acuity

One hundred seventy-two patients had longitudinal data for VA with a minimum follow-up of 12 months and a mean ± SD follow-up of 9.69 ± 9.09 years (range, 1.00–55.75 years). For the right eye, a significant difference (*P* < 0.001, paired *t* test) was found between mean ± SD VA of 0.36 ± 0.44 logMAR (Snellen equivalent, mean, 20/46) at baseline and 0.50 ± 0.57 logMAR (Snellen equivalent, mean, 20/63) at latest follow-up. This was also the case for the left eye (*P* < 0.001, paired *t* test), with a mean ± SD BCVA of 0.33 ± 0.39 logMAR (Snellen equivalent, mean, 20/43) at baseline and 0.43 ± 0.46 logMAR (Snellen equivalent, mean, 20/54) at latest follow-up. The mean annual progression rate was 0.013 logMAR (95% confidence interval, 0.004–0.022 logMAR) for the right eye (equates to 0.65 Early Treatment Diabetic Retinopathy Study (ETDRS) letters/year) and 0.009 logMAR (95% confidence interval, –0.002 to 0.020 logMAR) for the left eye (equates to 0.45 ETDRS letters/year).

### Longitudinal Analysis of Gass Stages

Longitudinal analysis of Gass stages was performed in 239 eyes from 124 patients with a minimum follow-up of 3 months. Mean ± SD age at baseline was 32.2 ± 21.3 years (range, 1.2–80.1 years), and mean ± SD follow-up was 8.3 ± 8.1 years (range, 0.3–43.1 years). Of 124 patients, 74 patients (59.7%) did not exhibit change in the Gass stage, with a mean ± SD follow-up of 6.4 ± 6.6 years. Gass stage changed in 50 patients in at least 1 eye, with a mean ± SD follow-up of 11.2 ± 9.4 years.

At baseline, 27 eyes (11.3%) were in the previtelliform stage (stage 1), which dropped to 21 eyes (9.2%) at last visit (mean ± SD follow-up, 6.5 ± 5.4 years). The vitelliform stage (stage 2) was observed in 71 eyes (29.7%) at baseline, with a decline to 37 eyes (15.5%) at last follow-up (mean ± SD follow-up, 9.8 ± 9.8 years). The pseudohypopyon stage (stage 3) was found in 24 eyes (10.0%) at baseline and in 19 eyes (8.0%) at last visit (mean ± SD follow-up, 6.3 ± 5.9 years). Vitelliruptive changes (stage 4) were diagnosed in 33 eyes (13.8%) at baseline, with an increase to 46 eyes (19.3%) at last visit (mean ± SD follow-up, 6.3 ± 5.7 years). Similarly, atrophic or fibrotic changes (stage 5) increased from 84 eyes (35.1%) at baseline to 115 eyes (48.1%) at last the follow-up (mean ± SD follow-up, 9.2 ± 8.6 years). In summary, a decrease in frequency of stages 1, 2, and 3 was found, with an increase in the frequency of stages 4 and 5 being found from baseline to the last visit (Fig 1B).

### Electrooculography

Electrooculography was available for 244 eyes from 122 patients. Two hundred twenty-three eyes (91.4%) exhibited a light peak-to-dark trough ratio of 1.5 or less or did not show any light rise, thereby meeting the diagnostic criteria for BVMD. Sixteen eyes (6.6%) exhibited a light peak-to-dark trough ratio of more than 1.5 and less than 1.85, and 5 eyes (2.0%) showed a light peak-to-dark trough ratio of 1.85 or more, which is considered the lower end of the normal range.

### Stratification According to Age at Onset

Based on the first time that BCVA was reduced to 0.2 logMAR or more (Snellen equivalent, 20/32), we separately assessed patients with adult-onset disease (≥ 18 years of age) and childhood-onset disease (< 18 years of age). Forty patients (22.5%) were classified as having childhood-onset disease and 138 patients (77.5%) were classified as having adult-onset disease. Visual acuity for both groups was plotted against age (Fig 2B), and linear regression did not reveal a significant difference between the lines of best fit (*P* = 0.09), although a trend of a slower decline of BCVA was found in the childhood-onset group compared with the patients in the adult-onset group.

In contrast, 21 patients (52.5%) in the childhood-onset group received a diagnosis of CNV, whereas 24 patients (17.4%) from the adult-onset group received a diagnosis of CNV, suggesting a lower rate of CNV in the adult-onset group (*z* = 4.50; *P* < 0.0001, chi-square test). A CNV diagnosis in the childhood-onset group was made at the mean ± SD age of 12.0 ± 4.8 years (range, 4.3–26.1 years), significantly lower (*t* = 6.5; *P* < 0.0001, unpaired *t* test with Welch’s correction) than in the adult-onset group, with mean ± SD age at diagnosis of 39.2 ± 17.5 years (range, 13.2–73.1 years).

### Genetic Characterization

**Figure 3:**
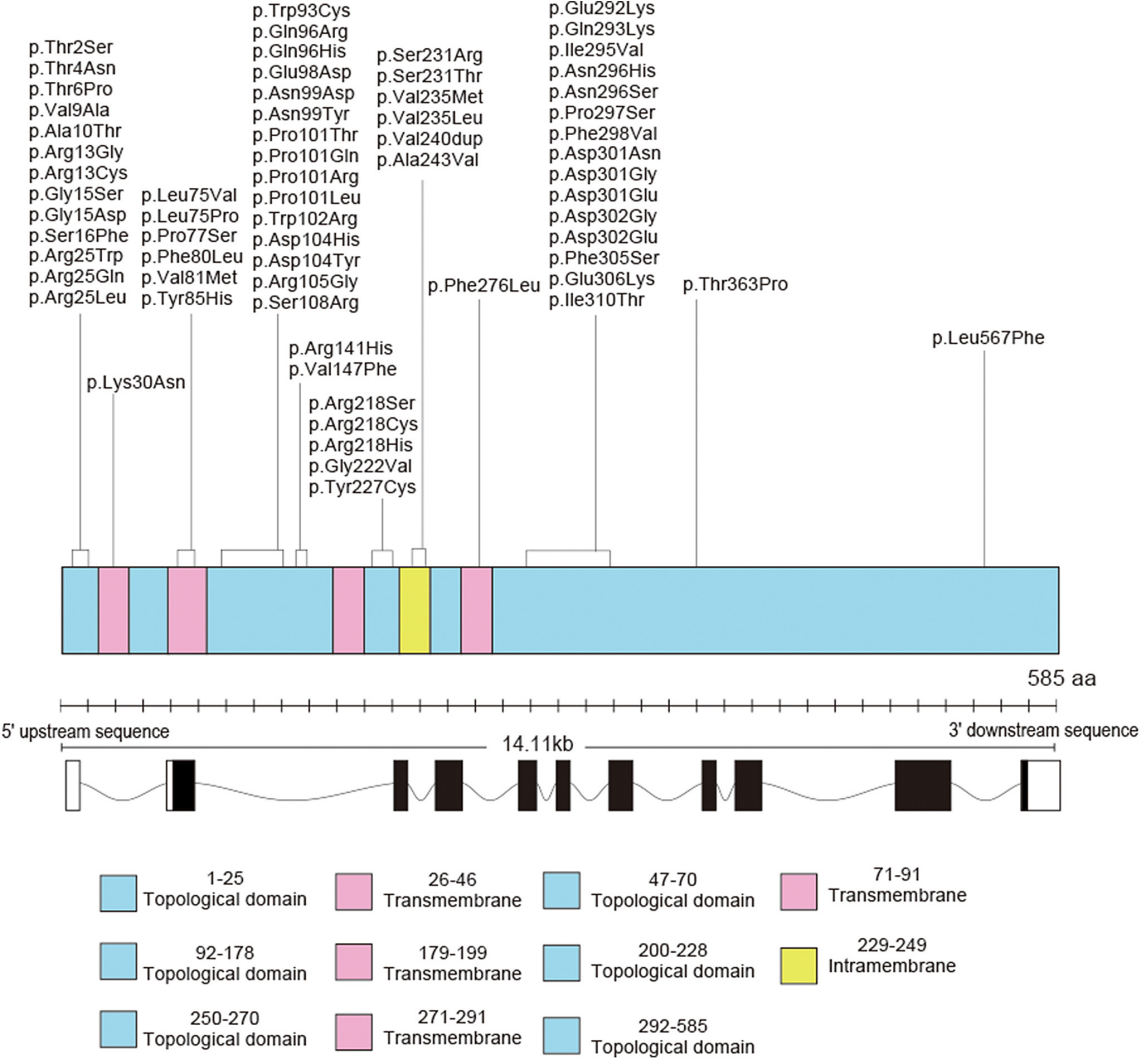
Schematic diagram showing the genetic and protein structure of *BEST1*. The positions of detected *BEST1* variants are illustrated, encompassing protein information (*BEST1*: identification, 076090; Uniprot; accessed January 2023).

In total, 69 monoallelic variants were identified in *BEST1*. Forty-seven variants were reported previously, and 22 variants were unreported previously. The variants comprised 64 missense variants, 1 frameshift deletion, 1 frameshift duplication, 1 in-frame duplication, and 2 intronic variants. Thirty-five recurrent variants were detected in multiple patients and 34 unique variants were detected in a single individual pedigree. Four variants were classified as pathogenic, 47 variants likely were pathogenic, and 18 variants were of uncertain significance. The localization of the identified *BEST1* variants in the gene domains is illustrated in Figure 3. The detailed results of in silico molecular genetic analysis are presented in Table S3 (available at www.aaojournal.org) and evolutionary conservation for the detected variants is shown in Figure S4 (available at www.aaojournal.org).

The most prevalent variants were c.652C>T, p.(Arg218Cys) (16/444 alleles; 3.6%); c.653G>A, p.(Arg218His) (12/444 alleles; 2.7%); c.728C>T, p.(Ala243Val) (11/444 alleles, 2.5%); c.892T>G, p.(Phe298Val) (8/444 alleles; 1.8%); c.37C>T, p.(Arg13Cys) (7/444 alleles; 1.6%); c.288G>C, p.(Gln96His) (7/444 alleles; 1.6%); c.914T>C, p.(Phe305Ser) (7/444 alleles; 1.6%); and c.90G>C, p.(Lys30Asn) (5/444 alleles; 11.2%).

### Genotype–Phenotype Correlation

The 3 most prevalent variants were analyzed for genotype–phenotype correlation: p.(Arg218Cys), p.(Arg218His), and p.(Ala243Val). The mean ± SD age at onset was 21.5 ± 15.5 years (range, 5.0–47.3 years) for p.(Arg218Cys), 28.2 ± 16.0 years (range, 7.3–58.8 years) for p.(Arg218His), and 50.6 ± 20.7 years (range, 13.5–84.8 years) for p.A243V. Multivariant analysis revealed a significant difference between the variants (*P* = 0.0007, analysis of variance) showing a later onset for p.(Ala243Val) compared with p.(Arg218Cys) (*P* = 0.0006, *t* test with Tukey correction) and p.(Arg218His) (*P* = 0.012, *t* test with Tukey correction).

Mean ± SD age-adjusted BCVA was 0.43 ± 0.35 logMAR (Snellen equivalent, mean, 20/54) for p.(Arg218Cys), 0.47 ± 0.43 logMAR (Snellen equivalent, mean, 20/59) for p.(Arg218His), and 0.13 ± 0.34 logMAR (Snellen equivalent, mean, 20/27) for p.(Ala243Val). Multivariant analysis revealed a significant difference among the variants (*P* = 0.01, analysis of variance), showing a better age-adjusted BCVA for p.(Ala243Val) compared with p.(Arg218Cys) (*P* = 0.03, *t* test with Tukey correction) and p.(Arg218His) (*P* = 0.01, *t* test with Tukey correction).

For patients harboring p.(Arg218Cys) (28 eyes; mean ± SD follow-up, 7.1 ± 7.0 years), the frequency of Gass stage 1 dropped from 21.4% at baseline to 14.4% at last visit, similarly observed for stage 2 with a drop from 14.2% to 10.7%. The occurrence of stage 3 rose from 10.7% to 17.9%, that of stage 4 declined from 17.8% to 10.7%, and that of stage 5 increased from 35.7% to 46.4%. Patients harboring p.(Arg218His) (20 eyes; mean ± SD follow-up, 2.9 ± 2.7 years) exhibited a stable frequency of Gass stage 1 of 5.0% at baseline and at last visit. Stage 2 declined from 45.0% to 30.0%, whereas stage 3 became more frequent from 10.0% to 20.0%. Stage 4 decreased from 10.0% to 0.0%, and stage 5 increased from 30.0% to 45.0%. In contrast to both of the above variants, patients harboring p.(Ala243Val) (16 eyes; mean ± SD follow-up, 7.6 ± 5.5 years) showed a stable and high frequency of Gass stage 1 of 25.0% at baseline and at last visit. Stage 2 occurred in 50% of the patients at baseline and fell to 12.5% at last visit. No patient was classified as having stage 3 disease at baseline, whereas at last follow-up, 6.25% of patients were classified as having stage 3 disease. The occurrence of stage 4 disease increased from 12.5% to 43.8%, whereas the low rate of stage 5 disease of 12.5% remained stable from baseline to the last visit. In summary, a higher frequency of stages 1 and 2 disease was found in patients with p.(Ala243Val) compared with patients with p.(Arg218Cys) and p.(Arg218His); whereas stage 5 disease occurred more frequently in the latter variants, corroborating the decreased severity of p.(Ala243Val) compared with p.(Arg218Cys) and p.(Arg218His).

## Discussion

The current study systematically described the detailed molecular, clinical, and morphologic characteristics associated with BVMD both cross-sectionally and longitudinally over a broad range of ages. This cohort of 222 patients from 141 families represents the largest published series to date to undergo detailed clinical characterization and genotype–phenotype investigation.

### Clinical Presentation

The clinical characteristics of this cohort largely are in keeping with those in reports from smaller series in the literature. Most of the patients demonstrated a deterioration of central vision at presentation, followed by referral because of incidental findings or a positive family history of BVMD. The reported mean BCVA of 0.35 logMAR at presentation (mean age, 26.8 years) is similar to that in a published Chinese cohort (n = 87; mean age, 31.8 years) with a mean BCVA of 0.42 logMAR.^13^ The correlation of age and BCVA at presentation, as well as the slow annual progression rate of visual deterioration of (< 1 ETDRS letter/year) found in this cohort, largely are in keeping with previous reports describing a worse BCVA in older patients, with a rather slow progression rate in earlier stages of the disease.^6^^,^^14^^,^^15^ Similarly, the increase of frequency of more advanced stages (vitelliruptive and atrophy or fibrosis) at last visit compared with baseline corroborates the existing literature.^6^^,^^15^^,^^16^ Unilateral presentation with morphologic changes limited to 1 eye occurred rarely in this cohort and was reported previously for *BEST1* variants causing BVMD, as well as for adult-onset vitelliform macular dystrophy caused by variants in *IMPG2*.^17^^,^^18^

### Refractive Error and Amblyopia

Most of the cohort (78.7% [185/235 eyes]) showed a refractive error in the hyperopic spectrum, which is a greatly higher proportion than the 4.8% reported in a population-based cohort and is in agreement with previous BVMD reports.^19^^,^^20^ During embryonic development, impaired retinal pigment epithelium function resulting from alterations in *BEST1* is anticipated to exert an impact on choroidal thickness and to disrupt scleral growth, causing the high rate of hyperopic refractive errors.^21^^,^^22^ Although retinal elevation resulting from subretinal deposits or fluid seems to be plausible as an additional reason for hyperopia, it has been reported that no correlation was found between the degree of hyperopia and vitelliform lesion height estimated by central subfield thickness.^19^ Furthermore, similar refractive errors in both eyes of individuals exhibiting asymmetric vitelliform lesions and persistence of hyperopia after regression of the subretinal lesions in flat atrophic retinas has been observed.^19^^,^^23^

The rate of amblyopia in this cohort (6.1% [13/214 patients]) is higher than the national average (3.6%) described in a cohort of children from the United Kingdom,^20^ which may be associated with the higher frequency of refractive errors in BVMD. These findings stress the importance of a consequent management of highly prevalent refractive errors in BVMD to avoid the development of amblyopia, in addition to the visual impairment derived from the retinal phenotype.

Similar to BVMD, autosomal recessive bestrophinopathy (ARB) usually presents with hyperopic refractive error, but it is associated more often with angle closure, with a previously reported rate of 28.6% compared with 2.8% in the present cohort.^24^ Alterations in *BEST1* have been implicated in a spectrum of impaired ocular development, including reports of nanophthalmus, microcornea, early-onset cataract, and posterior staphyloma.^25^ A reason for the higher rate of angle closure in ARB may be the distinct subcellular protein quality control, leading to different protein degradation processes in BVMD and ARB. For autosomal recessive variants, a rapid degradation in the endoplasmic reticulum has been observed, whereas dominant variants were able to escape endoplasmic reticulum-associated degradation, leading to slower disintegration via an endolysosomal pathway. This provides an explanation for the described dominant-negative effects of most genetic alterations causing BVMD, but also could explain the more severe phenotype of ARB because of the diminished protein levels resulting from rapid degeneration in patients with biallelic or compound heterozygous variants causing ARB.^26^

### Choroidal Neovascularization

The occurrence of CNV in 17.3% of this cohort (37/213 patients) is higher than most previously described rates of clinically diagnosed CNV, ranging from 1.7% to 9.0%.^6^^,^^15^^,^^27^ Of note, a recent study with OCT angiography showed substantially higher rates of CNV of 50.4%, suggesting systematic underdiagnosis of CNV in BVMD.^28^ This can be attributed to the difficulty in identifying CNV within BVMD because of subretinal fluid and preexisting subretinal deposits being a feature of the underlying disease and not being associated exclusively with CNV.^29^

Comparing the mean BCVA at last visit of eyes with a diagnosis of CNV and eyes without a diagnosis of CNV did not reveal a significant difference in this cohort (0.44 logMAR vs. 0.47 logMAR). Although it has been reported that patients often retain a relatively good BCVA after the occurrence of CNV,^27^^,^^29^ this finding also might be in keeping with the hypothesis of an underdiagnosis of CNV, especially in earlier stages of the disease, driving progression and leading to a high number of patients in more advanced stages with undetected CNV as a cause.^28^^,^^30^ This hypothesis also is supported by our finding that the rate of CNV diagnosis is lower in the adult-onset group (17.4%) compared with the rate in the childhood-onset group (52.5%).

Comparing the outcome of CNV treated with an anti-VEGF agent and without treatment with an anti-VEGF agent, we observed a mean BCVA at last follow-up of 0.28 logMAR in the treated group and 0.62 logMAR in the observed group. This beneficial effect of anti-VEGF treatment corroborates previously published research,^29^^,^^31^ but selection bias in the present cohort has to be considered, with some patients not receiving anti-VEGF treatment despite diagnosis of CNV because of, for example, severe atrophic or fibrotic changes, or both. Given the low number of injections needed per eye, the low rate of reported adverse events, and the beneficial outcome of treated patients in this cohort, we recommend administering anti-VEGF in patients with BVMD with secondary CNV in the presence of any active CNV and advising the patient of potential concurrent vision-limiting features such as subretinal fibrosis or atrophy that could limit BCVA recovery.

### Molecular Genetics

Sixty-nine *BEST1* variants, including 22 novel variants, were detected in the current large cohort study. More than 90% were missense variants, in keeping with findings in a Chinese cohort (32/37 variants [86.5%]),^13^ and these missense variants are located in the highly conserved N-terminal half of the protein, as described previously.^25^^,^^32^ These findings are consistent with the hypothesized disease mechanism of dominant-negative effects.^26^^,^^33^ Interestingly, some of the detected variants in the current BVMD study also were identified in ARB^34^: c.37C>T, p.(Arg13Cys); c.302C>T, p.(Pro101Leu); and c.889C>T, p.(Pro297Ser). Patients with biallelic *BEST1* variants also can exhibit a phenotype similar to BVMD, whereas those with the same variants in a heterozygous state may not manifest the same clinical phenotype.^35^ Reports exist of patients with a semidominant inheritance exhibiting severe BVMD or ARB phenotype caused by biallelic variants and mild BVMD by monoallelic variant in the same family.^36^^,^^37^ Further functional studies such as chloride conductance, cellular localization, and stability may reveal the exact functional effect and disease mechanism of each variant.^38^

The prevalent variants identified in the current study show different clinical characteristics. More severe phenotypes were observed for p.(Arg218Cys) and p.(Arg218His) and a milder phenotype for p.(Ala243Val), as previously reported for p.(Arg218Cys) in comparison with p.(Ala243Val) in a smaller series.^39^ Although p.(Ala243Val) is localized in the intramembrane domain of the protein, functional analyses showed intact trafficking of BEST1 to the plasma membrane. However, the chloride ion current has been found to be impaired, being 10% of wild-type. Furthermore, cotransfection of p.(Ala243Val) with wild-type did not impair the ion current of the wild-type channel in a dominant-negative way that has been described for other variants.^40^ Interestingly, for the most prevalent *BEST1* variant in this cohort, p.(Arg218Cys), mutant allele-specific gene editing restored calcium-activated chloride channel activity in human induced pluripotent stem cell-derived retinal pigment epithelium, indicating that gene augmentation therapy might be effective for these patients.^41^

### Study Limitations

Limitations of this study include the retrospective design, the absence of a control group, variability in follow-up duration, and lack of standardized protocol used for assessments. Different genetic testing protocols were applied, and familial segregation was not fully completed. This is the largest molecularly confirmed cohort to date, yet a larger cohort is needed to correlate structural and functional measures and to assess the progression rate of each *BEST1* variant reliably.

## Conclusions

This comprehensive analysis of clinical and genetic data of patients with BVMD contributes valuable insights for prognosis and genetic counseling and aids clinical trial design. Furthermore, the well-characterized cohort serves as a valuable resource for patient stratification in upcoming clinical trials, as well as further natural history investigations. The slow disease progression in this cohort indicates a broad therapeutic window before advancement into atrophic or fibrotic stages, especially for the milder variant p.A243V. Conversely, our identification of CNV incidence in young patients might underline the potential benefits of initiating treatment at a relatively early age.
