# Supplementary material for: Best Vitelliform Macular Dystrophy Natural History Study Report 1: Clinical Features and Genetic Findings
Source: Ophthalmology. 2024 Jul;131(7):845–54. doi: 10.1016/j.ophtha.2024.01.027 (PMC11932931; doi:10.1016/j.ophtha.2024.01.027)
Supplement: Supplemental Methods [file mmc1.pdf]

Supplemental methods

Genetic Analysis

All recruited patients were reassessed for their detected *BEST1* variants (PDE6A: Refseq Reference: (NM_004183.3, ENST00000378043.9; NP_004174.1; Uniprot ID: O76090-1). Sequence variant nomenclature was obtained according to the guidelines of the Human Genome Variation Society (HGVS) by using Mutalyzer 2.0. [1] Classification of all detected variants was also performed mainly based on the guidelines of the American College of Medical Genetics and Genomics (ACMG). [2,3]

In silico molecular modeling was conducted. Minor allele frequency for the identified variants in the general population was assessed in the Genome Aggregation Database (gnomAD) datasets. The population data and general coverage by whole exome sequence were also provided with the gnomAD database. General prediction scores were further calculated using MutationTaster, FATHMM, CADD, and REVEL. Functional prediction was performed employing SIFT, PROVEAN, and Polyphen 2. Human splicing finder 3.0 was applied for splicing defects prediction. Mammalian (PhyloP30way and PhastCons30way) and vertebrate (PhyloP100way and PhastCons100way) conservation were also investigated. The previously reported variants were surveyed with the HGMD database and ClinVar database (accessed in January 2023).

The evolutionary conservation was assessed by multiple alignments of species of the *RP1L1* gene sequence using the Clustal Omega program (https://www.ebi.ac.uk/Tools/msa/clustalo/).

1. Lefter M, Vis JK, Vermaat M, den Dunnen JT, Taschner PEM, Laros JFJ. Mutalyzer 2: next generation HGVS nomenclature checker. *Bioinformatics*. 2021;37(18):2811-2817. doi:10.1093/bioinformatics/btab051

2. Richards S, Aziz N, Bale S, et al. Standards and guidelines for the interpretation of sequence variants: a joint consensus recommendation of the American College of Medical Genetics and Genomics and the Association for Molecular Pathology. *Genet Med*. 2015;17(5):405-424. doi:10.1038/gim.2015.30

3. Abou Tayoun AN, Pesaran T, DiStefano MT, et al. Recommendations for interpreting the loss of function PVS1 ACMG/AMP variant criterion. *Hum Mutat*. 2018;39(11):1517-1524. doi:10.1002/humu.23626
