# Supplementary material for: Best Vitelliform Macular Dystrophy Natural History Study Report 1: Clinical Features and Genetic Findings
Source: Ophthalmology. 2024 Jul;131(7):845–54. doi: 10.1016/j.ophtha.2024.01.027 (PMC11932931; doi:10.1016/j.ophtha.2024.01.027)
Supplement: Figure S4 [file mmc2.pdf]

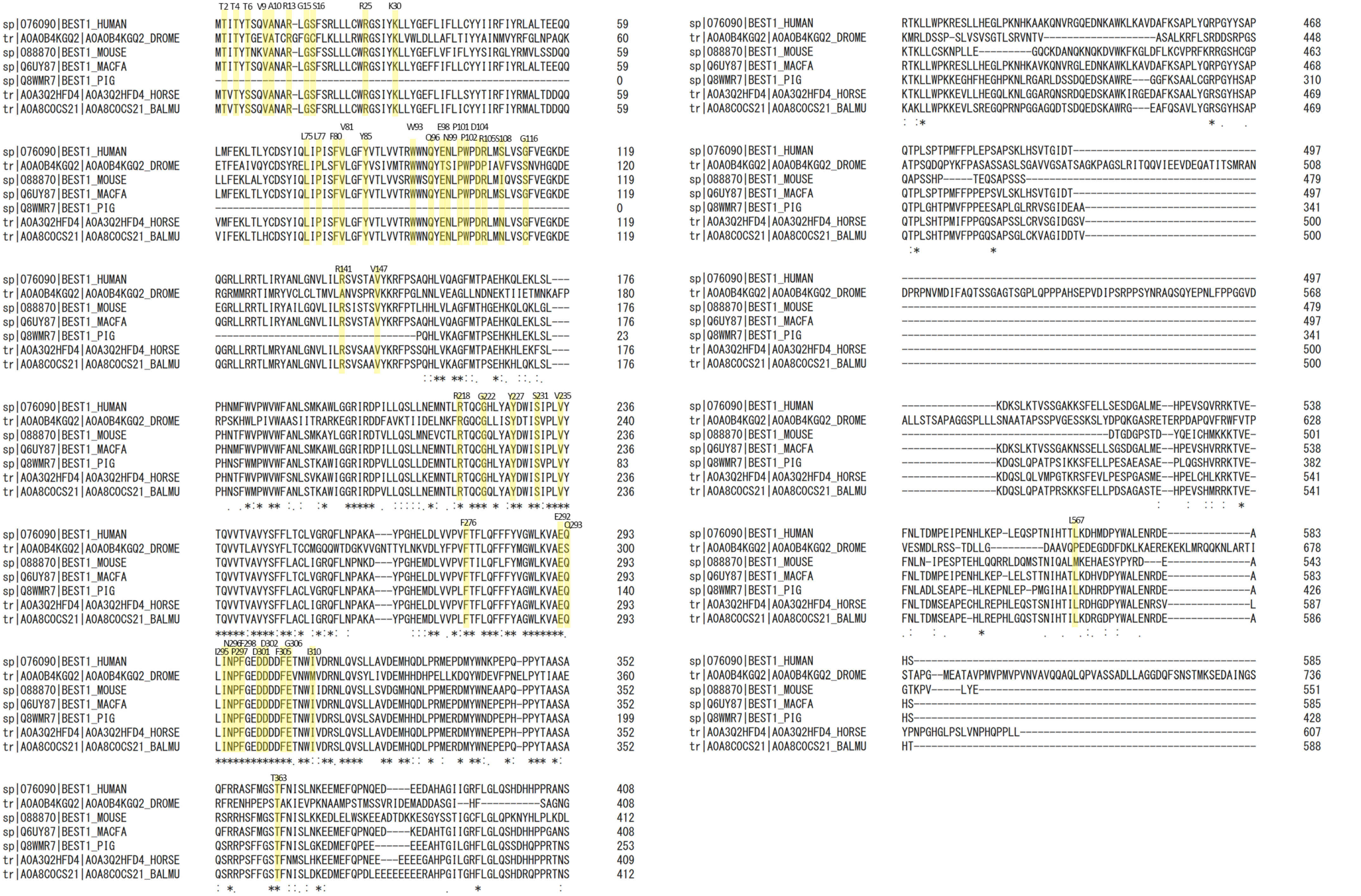

Supplementary Figure 1. Evolutionary conservation for the detected BEST1 variants.

Multiple alignments of seven species of the *BEST1* gene are demonstrated. The alignment was performed with the Clustal Omega program (https://www.ebi.ac.uk/Tools/msa/clustalo/), and the amino acid-sequence alignment was numbered in accordance with the Homo sapiens *BEST1* sequence (ENST00000378043.9). The detected variants are highlighted with yellow background. An asterisk indicates high conservation across the seven species.
