# Supplementary material for: Best Vitelliform Macular Dystrophy Natural History Study Report 1: Clinical Features and Genetic Findings
Source: Ophthalmology. 2024 Jul;131(7):845–54. doi: 10.1016/j.ophtha.2024.01.027 (PMC11932931; doi:10.1016/j.ophtha.2024.01.027)
Supplement: Table S2 [file mmc3.pdf]

Supplementary Table S2: List of peripheral retinal findings for 422 eyes.

Retinal Finding	Eyes, n=	%
Peripapillary hyperfluorescent area (superiotemporal)	34	8.1
Multiple yellow hyperautofluorescent spots in middle periphery	24	5.7
Pavingstones	10	2.4
White without pressure	6	1.4
Circumscribed atrophic changes nasally	2	0.5
Drusen in middle periphery	2	0.5
Hyperpigmented lesion	2	0.5
Irregular speckled pattern of autofluorescence at superior arcade	2	0.5
Lattice degeneration	2	0.5
Multiple atrophic patches around superior arcade	2	0.5
Multiple hyperpigmented spots	2	0.5
Multiple vitelliform lesions around superior arcade	2	0.5
Myelinated nerve fibers	2	0.5
Retinal pigment epithelium changes with yellowish hyperautofluorescent spots	2	0.5
Scars after cryoretinopexy	2	0.5
Vitelliform lesion above the disc	2	0.5
Vitelliform lesion nasal periphery	2	0.5
Speckled retinal pigment epithelium changes in periphery	2	0.5
Atrophic patch at superior arcade	1	0.2
Branch retinal vein occlusion with intraretinal hemorrhages and subretinal fluid	1	0.2
Buckle	1	0.2
Laser scars around retinal tear	1	0.2
Multiple hyperautofluorescent dots superior vascular arcade	1	0.2
Peripheral telangiectatic changes	1	0.2
Retinoschisis	1	0.2
Retinal pigment epithelium changes with hyperpigmented hyperautofluorescent spots	1	0.2
Single vitelliform lesion at superior arcade	1	0.2
Small atrophic patch and hyperpigmentation	1	0.2
Small yellowish deposits	1	0.2
Speckled retinal pigment epithelium changes in periphery	1	0.2
Vitelliform lesion above disc	1	0.2
None documented	317	75.1
