# Supplementary material for: Best Vitelliform Macular Dystrophy Natural History Study Report 1: Clinical Features and Genetic Findings
Source: Ophthalmology. 2024 Jul;131(7):845–54. doi: 10.1016/j.ophtha.2024.01.027 (PMC11932931; doi:10.1016/j.ophtha.2024.01.027)
Supplement: Table S3 [file mmc4.pdf]

Variant No	Patient/family number	Nucleotide change (NM_001030311.2)	Position	Coding impact	Amino acid change	Location	dbSNP ID	ClinVar ID	Segregation	ACMG Classification: Verdict	Factor 1	Factor 2	Factor 3	Factor 4	Factor 5	Factor 6	Factor 7
1	1, 2	NM_004183.4:c.5C>G	61719283	Missense	NP_004174.1:p.Thr2Ser	exon 2 of 11 position 41 of 188 (coding)	NA	866344	unknown/ not tested	Likely pathogenic	PM2	PM5	PP1	PP3	PP5		
2	4	NM_004183.4:c.11C>A	61719289	Missense	NP_004174.1:p.Thr4Asn	exon 2 of 11 position 47 of 188 (coding)	rs368383940	NA	1 parent tested negative, other one unknown/not tested	VUS	PM2	PM5	PP3				
3	5	NM_004183.4:c.16A>C	61719294	Missense	NP_004174.1:p.Thr6Pro	exon 2 of 11 position 52 of 188 (coding)	rs28940275	2732	unknown/ not tested	Likely pathogenic	PM2	PM5	PP1	PP3	PP5		
4	6,7, 175, 176	NM_004183.4:c.26T>C	61719304	Missense	NP_004174.1:p.Val9Ala	exon 2 of 11 position 62 of 188 (coding)	rs281865205	99700	positive parent/ sibling	Likely pathogenic	PM2	PM5	PP1	PP3	PP5		
5	7,8,9	NM_004183.4:c.28G>A	61719306	Missense	NP_004174.1:p.Ala10Thr	exon 2 of 11 position 64 of 188 (coding)	rs281865206	99706	unknown/ not tested	Likely pathogenic	PM2	PM5	PP1	PP3	PP5		
6	10	NM_004183.4:c.37C>G	61719315	Missense	NP_004174.1:p.Arg13Gly	exon 2 of 11 position 73 of 188 (coding)	NA	444253	unknown/ not tested	Likely pathogenic	PM2	PM5	PP1	PP3	PP5		
7	11, 12, 13, 14, 15, 16, 17	NM_004183.4:c.37C>T	61719315	Missense	NP_004174.1:p.Arg13Cys	exon 2 of 11 position 73 of 188 (coding)	NA	279701	positive parent/ sibling	Likely pathogenic	PM2	PM5	PP1	PP3	PP5		
8	18	NM_004183.4:c.43G>A	61719321	Missense	NP_004174.1:p.Gly15Ser	exon 2 of 11 position 79 of 188 (coding)	NA	NA	positive parent/ sibling	VUS	PM2	PM5	PP3				
9	19,20	NM_004183.4:c.44G>A	61719322	Missense	NP_004174.1:p.Gly15Asp	exon 2 of 11 position 80 of 188 (coding)	NA	429731	unknown/ not tested	Likely pathogenic	PM2	PM5	PP1	PP3	PP5		
10	21,22,23,24	NM_004183.4:c.47C>T	61719325	Missense	NP_004174.1:p.Ser16Phe	exon 2 of 11 position 83 of 188 (coding)	rs281865210	99723	1 positive sibling	Likely pathogenic	PM2	PM5	PP1	PP3	PP5		
11	25	NM_004183.4:c.73C>T	61719351	Missense	NP_004174.1:p.Arg25Trp	exon 2 of 11 position 109 of 188 (coding)	rs281865214	99751	unknown/ not tested	Likely pathogenic	PM2	PM5	PP1	PP3	PP5		
12	26	NM_004183.4:c.74G>A	61719352	Missense	NP_004174.1:p.Arg25Gln	exon 2 of 11 position 110 of 188 (coding)	rs281865215	99752	unknown/ not tested	Likely pathogenic	PM2	PM5	PP1	PP3	PP5		
13	27, 28, 29	NM_004183.4:c.74G>T	61719352	Missense	NP_004174.1:p.Arg25Leu	exon 2 of 11 position 110 of 188 (coding)	NA	NA	positive parent/ sibling	Likely pathogenic	PM2	PM5	PP1	PP3	PP5		
14	30, 31, 32, 33, 34	NM_004183.4:c.90G>C	61719368	Missense	NP_004174.1:p.Lys30Asn	exon 2 of 11 position 126 of 188 (coding)	NA	NA	unknown/ not tested	Likely pathogenic	PM2	PM5	PP1	PP3	PP5		
15	35, 173	NM_004183.4:c.223C>G	61722649	Missense	NP_004174.1:p.Leu75Val	exon 3 of 11 position 71 of 95 (coding)	NA	NA	positive parent/ sibling	Likely pathogenic	PM2	PM5	PP1	PP3	PP5		
16	36, 37, 38	NM_004183.4:c.224T>C	61722650	Missense	NP_004174.1:p.Leu75Pro	exon 3 of 11 position 71 of 95 (coding)	NA	1375926	1 positive sibling	Likely pathogenic	PM2	PM5	PP1	PP3	PP5		
17	39	NM_004183.4:c.229C>T	61722655	Missense	NP_004174.1:p.Pro77Ser	exon 3 of 11 position 77 of 95 (coding)	NA	NA	unknown/ not tested	Likely pathogenic	PS3	PM2	PP3	PP5			
18	40	NM_004183.4:c.240C>G	61722666	Missense	NP_004174.1:p.Phe80Leu	exon 3 of 11 position 88 of 95 (splicing, coding)	NA	1459557	1 positive sibling	Likely pathogenic	PM2	PM5	PP1	PP3			
19	41	NM_004183.4:c.241G>A	61722667	Missense	NP_004174.1:p.Val81Met	exon 3 of 11 position 89 of 95 (splicing, coding)	NA	548451	positive parent/ sibling	Likely pathogenic	PM2	PM5	PP1	PP3	PP5		
20	42, 43	NM_004183.4:c.253T>C	61723195	Missense	NP_004174.1:p.Tyr85His	exon 4 of 11 position 6 of 234 (splicing, coding)	rs28940274	2728	unknown/ not tested	VUS	PM2	PP3	PP5				
21	44	NM_004183.4:c.279G>T	61723221	Missense	NP_004174.1:p.Trp93Cys	exon 4 of 11 position 32 of 234 (coding)	NA	NA	1 parent tested negative, other one ukn/untested	Pathogenic	PS3	PM2	PM5	PP3	PP5		
22	45	NM_004183.4:c.287A>G	61723229	Missense	NP_004174.1:p.Gln96Arg	exon 4 of 11 position 40 of 234 (coding)	NA	635998	unknown/ not tested	Pathogenic	PS3	PM2	PM5	PP3	PP5		
23	46, 47, 48, 49, 50, 52, 53	NM_004183.4:c.288G>C	61723230	Missense	NP_004174.1:p.Gln96His	exon 4 of 11 position 41 of 234 (coding)	rs281865226	99705	2 positive siblings	Likely pathogenic	PM2	PM5	PP1	PP3	PP5		
24	54	NM_004183.4:c.294G>T	61723236	Missense	NP_004174.1:p.Glu98Asp	exon 4 of 11 position 47 of 234 (coding)	NA	NA	unknown/ not tested	VUS	PM2	PM5	PP3				
25	55	NM_004183.4:c.295A>G	61723237	Missense	NP_004174.1:p.Asn99Asp	exon 4 of 11 position 48 of 234 (coding)	NA	NA	unknown/ not tested	Likely pathogenic	PM2	PM5	PP1	PP3	PP5		
26	56	NM_004183.4:c.295A>T	61723237	Missense	NP_004174.1:p.Asn99Tyr	exon 4 of 11 position 48 of 234 (coding)	NA	NA	unknown/ not tested	Likely pathogenic	PM2	PM5	PP3	PP5			
27	57	NM_004183.4:c.301C>A	61723243	Missense	NP_004174.1:p.Pro101Thr	exon 4 of 11 position 54 of 234 (coding)	rs281865229	99710	unknown/ not tested	Likely pathogenic	PM2	PM5	PP1	PP3	PP5		
28	58	NM_004183.4:c.302C>A	61723244	Missense	NP_004174.1:p.Pro101Gln	exon 4 of 11 position 55 of 234 (coding)	NA	NA	unknown/ not tested	VUS	PM2	PM5	PP3				
29	59	NM_004183.4:c.302C>G	61723244	Missense	NP_004174.1:p.Pro101Arg	exon 4 of 11 position 55 of 234 (coding)	NA	870723	unknown/ not tested	VUS	PM2	PM5	PP3				
30	60, 61, 62	NM_004183.4:c.302C>T	61723244	Missense	NP_004174.1:p.Pro101Leu	exon 4 of 11 position 55 of 234 (coding)	rs374517178	877528	unknown/ not tested	VUS	PM2	PM5	PP3				
31	63	NM_004183.4:c.304T>C	61723246	Missense	NP_004174.1:p.Trp102Arg	exon 4 of 11 position 57 of 234 (coding)	rs281865230	99711	unknown/ not tested	Likely pathogenic	PM2	PM5	PP3	PP5			
32	64, 65	NM_004183.4:c.310G>C	61723252	Missense	NP_004174.1:p.Asp104His	exon 4 of 11 position 63 of 234 (coding)	rs281865231	99712	unknown/ not tested	Likely pathogenic	PM2	PM5	PP1	PP3	PP5		
33	66	NM_004183.4:c.310G>T	61723252	Missense	NP_004174.1:p.Asp104Tyr	exon 4 of 11 position 63 of 234 (coding)	NA	NA	unknown/ not tested	VUS	PM2	PM5	PP3				
34	67, 68	NM_004183.4:c.313C>G	61723255	Missense	NP_004174.1:p.Arg105Gly	exon 4 of 11 position 66 of 234 (coding)	NA	932077	positive parent/ sibling	Likely pathogenic	PM2	PM5	PP1	PP3	PP5		
35	69, 70, 71	NM_004183.4:c.324C>G	61723266	Missense	NP_004174.1:p.Ser108Arg	exon 4 of 11 position 77 of 234 (coding)	NA	544680	positive parent/ sibling	VUS	PM2	PP1	PP3	PP5			
36	72	NM_004183.4:c.345delA	61723286	LoF	NP_004174.1:p.Gly116Alafs*48	exon 4 of 11 position 98 of 234 (coding, NMD)	NA	NA	positive parent/ sibling	Pathogenic	PVS1	PP1	PM2				
37	74	NM_004183.4:c.439G>T	61723381	Missense	NP_004174.1:p.Val147Phe	exon 4 of 11 position 192 of 234 (coding)	NA	NA	unknown/ not tested	VUS	PM2	PP3					
38	75, 76, 77	NM_004183.4:c.652C>A	61724874	Missense	NP_004174.1:p.Arg218Ser	exon 6 of 11 position 16 of 78 (coding)	rs281865238	99734	positive parent/ sibling	Likely pathogenic	PM2	PM5	PP1	PP3	PP5		
39	78, 79, 80, 81, 82, 83, 84, 85, 86, 87, 88, 89, 90, 91, 92, 93	NM_004183.4:c.652C>T	61724874	Missense	NP_004174.1:p.Arg218Cys	exon 6 of 11 position 16 of 78 (coding)	rs281865238	99735	1 positive sibling	Likely pathogenic	PM2	PM5	PP1	PP3	PP5		
40	94, 95, 96, 97, 98, 99, 100, 101, 102, 103, 104, 105	NM_004183.4:c.653G>A	61724875	Missense	NP_004174.1:p.Arg218His	exon 6 of 11 position 17 of 78 (coding)	rs281865239	99736	positive parent/ sibling	Likely pathogenic	PM2	PM5	PP1	PP3	PP5		
41	106	NM_004183.4:c.665G>T	61724887	Missense	NP_004174.1:p.Gly222Val	exon 6 of 11 position 29 of 78 (coding)	rs281865241	99739	unknown/ not tested	Likely pathogenic	PM2	PM5	PP3	PP5			
42	107	NM_004183.4:c.680A>G	61724902	Missense	NP_004174.1:p.Tyr227Cys	exon 6 of 11 position 44 of 78 (coding)	rs267606677	2749	unknown/ not tested	Likely pathogenic	PM2	PM5	PP3	PP5			
43	108, 109	NM_004183.4:c.691A>C	61724913	Missense	NP_004174.1:p.Ser231Arg	exon 6 of 11 position 55 of 78 (coding)	NA	NA	positive parent/ sibling	Likely pathogenic	PM2	PM5	PP1	PP3	PP5		
44	110, 178	NM_004183.4:c.692G>C	61724914	Missense	NP_004174.1:p.Ser231Thr	exon 6 of 11 position 56 of 78 (coding)	NA	NA	positive parent/ sibling	Likely pathogenic	PM2	PM5	PP1	PP3	PP5		
45	111	NM_004183.4:c.703G>A	61724925	Missense	NP_004174.1:p.Val235Met	exon 6 of 11 position 67 of 78 (coding)	rs281865245	99744	both parents tested negative	Likely pathogenic	PM2	PM5	PP3	PP5			
46	112, 113	NM_004183.4:c.703G>T	61724925	Missense	NP_004174.1:p.Val235Leu	exon 6 of 11 position 67 of 78 (coding)	NA	870724	unknown/ not tested	Likely pathogenic	PM2	PM5	PP1	PP3	PP5		
47	114	NM_004183.4:c.718_720dupGTG	61725617	Other	NP_004174.1:p.Val240dup	exon 7 of 11 before position 7 of 153 (splicing, coding)	NA	NA	positive parent/ sibling	VUS	PM2	PM4	PP1				
48	115-125	NM_004183.4:c.728C>T	61725631	Missense	NP_004174.1:p.Ala243Val	exon 7 of 11 position 14 of 153 (coding)	rs28940570	2737	positive parent/ sibling	Likely pathogenic	PM2	PM5	PP1	PP3	PP5		
49	126, 127, 239, 129	NM_004183.4:c.826T>C	61725729	Missense	NP_004174.1:p.Phe276Leu	exon 7 of 11 position 112 of 153 (coding)	NA	NA	positive parent/ sibling	VUS	PM2	PP1	PP3	PP5			
50	130, 131, 132	NM_004183.4:c.874G>A	61726976	Missense	NP_004174.1:p.Glu292Lys	exon 8 of 11 position 7 of 81 (splicing, coding)	NA	265047	positive parent/ sibling	Likely pathogenic	PM2	PM5	PP1	PP3	PP5		
51	133	NM_004183.4:c.877C>A	61726979	Missense	NP_004174.1:p.Gln293Lys	exon 8 of 11 position 10 of 81 (splicing, coding)	rs281865250	99758	unknown/ not tested	Likely pathogenic	PM2	PM5	PP3	PP5			
52	134, 135, 137	NM_004183.4:c.883A>G	61726985	Missense	NP_004174.1:p.Ile295Val	exon 8 of 11 position 16 of 81 (coding)	rs777320382	1303124	unknown/ not tested	Likely pathogenic	PM2	PM5	PP1	PP3	PP5		
53	138, 140, 141	NM_004183.4:c.886A>C	61726988	Missense	NP_004174.1:p.Asn296His	exon 8 of 11 position 19 of 81 (coding)	rs281865254	99763	positive parent/ sibling	Likely pathogenic	PM2	PM5	PP1	PP3	PP5		
54	142	NM_004183.4:c.887A>G	61726989	Missense	NP_004174.1:p.Asn296Ser	exon 8 of 11 position 20 of 81 (coding)	rs281865255	99764	unknown/ not tested	Likely pathogenic	PM2	PM5	PP1	PP3	PP5		
55	143, 144	NM_004183.4:c.889C>T	61726991	Missense	NP_004174.1:p.Pro297Ser	exon 8 of 11 position 22 of 81 (coding)	rs1805143	99766	unknown/ not tested	Likely pathogenic	PM2	PM5	PP1	PP3	PP5		
56	145, 146, 147, 148, 149, 150, 151, 152	NM_004183.4:c.892T>G	61726994	Missense	NP_004174.1:p.Phe298Val	exon 8 of 11 position 25 of 81 (coding)	NA	NA	positive parent/ sibling	Likely pathogenic	PM2	PM5	PP1	PP3	PP5		
57	153	NM_004183.4:c.901G>A	61727003	Missense	NP_004174.1:p.Asp301Asn	exon 8 of 11 position 34 of 81 (coding)	rs281865259	99771	positive parent/ sibling	Likely pathogenic	PM2	PM5	PP1	PP3	PP5		
58	154, 155, 156	NM_004183.4:c.902A>G	61727004	Missense	NP_004174.1:p.Asp301Gly	exon 8 of 11 position 35 of 81 (coding)	NA	1457091	positive parent/ sibling	Likely pathogenic	PM2	PM5	PP1	PP3	PP5		
59	157, 158, 159	NM_004183.4:c.903T>A	61727005	Missense	NP_004174.1:p.Asp301Glu	exon 8 of 11 position 36 of 81 (coding)	NA	NA	positive parent/ sibling	Likely pathogenic	PM2	PM5	PP1	PP3	PP5		
60	160	NM_004183.4:c.905A>G	61727007	Missense	NP_004174.1:p.Asp302Gly	exon 8 of 11 position 38 of 81 (coding)	rs281865263	99775	both parents tested negative	Likely pathogenic	PM2	PM5	PP3	PP5			
61	161	NM_004183.4:c.906T>G	61727008	Missense	NP_004174.1:p.Asp302Glu	exon 8 of 11 position 39 of 81 (coding)	NA	NA	unknown/ not tested	VUS	PM2	PM5	PP3				
62	162, 163, 164, 165, 174, 190, 192	NM_004183.4:c.914T>C	61727016	Missense	NP_004174.1:p.Phe305Ser	exon 8 of 11 position 47 of 81 (coding)	rs281865265	99778	positive parent/ sibling	Likely pathogenic	PM2	PM5	PP1	PP3	PP5		
63	193, 194, 198	NM_004183.4:c.916G>A	61727018	Missense	NP_004174.1:p.Glu306Lys	exon 8 of 11 position 49 of 81 (coding)	NA	NA	1 positive sibling	Likely pathogenic	PM2	PM5	PP1	PP3	PP5		
64	200	NM_004183.4:c.929T>C	61727031	Missense	NP_004174.1:p.Ile310Thr	exon 8 of 11 position 62 of 81 (coding)	rs281865271	99784	unknown/ not tested	VUS	PM2	PP1	PP3	PP5			
65	201	NM_004183.4:c.948+18G>C	61727068	Other	NA	intron 8 of 10 position 18 of 313 (intronic)	NA	NA	unknown/ not tested	VUS	PM2	BP4					
66	209	NM_004183.4:c.949-17_949-16delCT	61727346	Other	NA	intron 8 of 10 position 297-298 of 313 (intronic)	rs774668020	1078189	unknown/ not tested	VUS	PM2	BP4					
67	215	NM_004183.4:c.1087A>C	61727502	Missense	NP_004174.1:p.Thr363Pro	exon 9 of 11 position 139 of 152 (coding)	rs147228028	843874	positive parent/ sibling	VUS	PM2	PP3					
68	223, 224, 225	NM_004183.4:c.1441dupC	61730063	LoF	NP_004174.1:p.Leu481Profs*34	exon 10 of 11 before position 342 of 639 (coding, NMD)	NA	NA	positive parent/ sibling	Pathogenic	PVS1	PM2	PP1				
69	227	NM_004183.4:c.1699C>T	61730325	Missense	NP_004174.1:p.Leu567Phe	exon 10 of 11 position 599 of 639 (coding)	rs148060787	99686	unknown/ not tested	VUS	PM2						

Sequence variant nomenclature was obtained according to the guidelines of the Human Genome Variation Society (HGVS) by using Mutalyzer (https://mutalyzer.nl/). All detected variants were analysed using two general prediction programs and three functional prediction programs; Mutation Taster (http://www.mutationtaster.org/), FATHMM (http://fathmm.biocompute.org.uk/9), SIFT (https://www.sift.co.uk/), PROVEAN (http://provean.jcvi.org/index.php), and Polyphen 2 (http://genetics.bwh.harvard.edu/pph2/). Minor allele frequency for the identified variants in the general population was assessed in the Genome Aggregation Database (gnomAD) datasets(https://gnomad.broadinstitute.org/). The population data and general coverage were also provided with the gnomAD database. Evolutionary conservation scores were calculated referring to the UCSC database (https://genome.ucsc.edu/index.html). Classification of predictions by the American College of Medical Genetics and Genomics (ACMG) was also applied for all detected variants.
Genome reference; Hg19 Transcript; NM_004183.3, ENST00000378043.9 or NM_001139443.2, ENST00000449131
